# Supplementary material for: Using Kinetic Modelling to Infer Adaptations in Saccharomyces cerevisiae Carbohydrate Storage Metabolism to Dynamic Substrate Conditions
Source: Metabolites. 2023 Jan 5;13(1):88. doi: 10.3390/metabo13010088 (PMC9862193; doi:10.3390/metabo13010088)
Supplement: Supplementary file 1 [file metabolites-13-00088-s001.zip › metabolites-1971060-supplementary/supplementary_materials/File S1/Rate_equations.pdf]

Rate Equations

The following kinetic expressions were used:

$$v_{\mathrm{GLT}} = \frac{V_m \left(\mathrm{GLCo} - \frac{\mathrm{GLCi}}{K_{\mathrm{eq}}}\right)}{K_m \left(\frac{\mathrm{GLCi}}{K_m} + \frac{\mathrm{GLCo}}{K_m} + \frac{\mathrm{GLCi}\,\mathrm{GLCo}\,K_i}{{K_m}^2} + 1\right)} \tag{1}$$

$$v_{\mathrm{HXK}} = \frac{V_m \left(\mathrm{ATP}\,\mathrm{GLCi} - \frac{\mathrm{ADP}\,\mathrm{G6P}}{K_{\mathrm{eq}}}\right)}{K_{m,\mathrm{glc}}\,K_{m,\mathrm{atp}} \left(\frac{\mathrm{ADP}}{K_{m,\mathrm{adp}}} + \frac{\mathrm{ATP}}{K_{m,\mathrm{atp}}} + 1\right) \left(\frac{\mathrm{G6P}}{K_{m,\mathrm{g6p}}} + \frac{\mathrm{GLCi}}{K_{m,\mathrm{glc}}} + \frac{\mathrm{T6P}}{K_{i,\mathrm{t6p}}} + 1\right)} \tag{2}$$

$$v_{\mathrm{PGM1}} = \frac{V_m \left(\mathrm{G1P} - \frac{\mathrm{G6P}}{K_{\mathrm{eq}}}\right)}{K_{m,\mathrm{G1P}} \left(\frac{\mathrm{G1P}}{K_{m,\mathrm{G1P}}} + \frac{\mathrm{G6P}}{K_{m,\mathrm{G6P}}} + 1\right)} \tag{3}$$

$$v_{\mathrm{TPS1}} = \frac{V_m\,\mathrm{F6P}\,\mathrm{G6P}\,\mathrm{UDPGLC}}{K_{m,\mathrm{G6P}}\,K_{m,\mathrm{UDPGLC}} \left(\frac{\mathrm{G6P}}{K_{m,\mathrm{G6P}}} + 1\right) \left(\frac{\mathrm{PI}}{K_{i,\mathrm{PI}}} + 1\right) \left(\frac{\mathrm{UDPGLC}}{K_{m,\mathrm{UDPGLC}}} + 1\right) \left(\mathrm{F6P} + K_{m,\mathrm{F6P}}\right)} \tag{4}$$

$$v_{\mathrm{TPS2}} = \frac{V_m\,\mathrm{PI}\,\mathrm{T6P}}{\mathrm{PI}\,\left(K_{m,\mathrm{T6P}} + \mathrm{T6P}\right) + K_{i,\mathrm{PI}}\,K_{m,\mathrm{T6P}}} \tag{5}$$

$$v_{\mathrm{NTH1}} = \frac{V_m\,\mathrm{TRE}}{K_{m,\mathrm{TRE}} \left(\frac{\mathrm{TRE}}{K_{m,\mathrm{TRE}}} + 1\right)} \tag{6}$$

$$v_{\mathrm{PGI}} = \frac{V_m \left(\mathrm{G6P} - \frac{\mathrm{F6P}}{K_{\mathrm{eq}}}\right)}{K_{m,\mathrm{g6p}} \left(\frac{\mathrm{F6P}}{K_{m,\mathrm{f6p}}} + \frac{\mathrm{G6P}}{K_{m,\mathrm{g6p}}} + 1\right)} \tag{7}$$

$$v_{\mathrm{PFK}} = \frac{V_m\,R\,g_R\,\mathrm{lam}_1\,\mathrm{lam}_2}{R^2 + L\,T^2} \tag{8}$$

$$\mathrm{lam}_1 = \frac{\mathrm{F6P}}{K_{R,\mathrm{F6P}}} \tag{9}$$

$$\mathrm{lam}_2 = \frac{\mathrm{ATP}}{K_{R,\mathrm{ATP}}} \tag{10}$$

$$R = \mathrm{lam}_1\,\mathrm{lam}_2 + g_R\,\mathrm{lam}_1\,\mathrm{lam}_2 + 1 \tag{11}$$

$$T = c_{\mathrm{ATP}}\,\mathrm{lam}_2 + 1 \tag{12}$$

$$L = \frac{L_0 \left(\frac{\mathrm{AMP}\,c_{i,\mathrm{AMP}}}{K_{\mathrm{AMP}}} + 1\right)^2 \left(\frac{\mathrm{ATP}\,c_{i,\mathrm{ATP}}}{K_{\mathrm{ATP}}} + 1\right)^2 \left(\frac{\mathrm{F26bP}\,c_{i,\mathrm{F26bp}}}{K_{\mathrm{F26bP}}} + \frac{\mathrm{FBP}\,c_{i,\mathrm{FBP}}}{K_{\mathrm{FBP}}} + 1\right)}{\left(\frac{\mathrm{AMP}}{K_{\mathrm{AMP}}} + 1\right)^2 \left(\frac{\mathrm{ATP}}{K_{\mathrm{ATP}}} + 1\right)^2 \left(\frac{\mathrm{F26bP}}{K_{\mathrm{F26bP}}} + \frac{\mathrm{FBP}}{K_{\mathrm{FBP}}} + 1\right)} \tag{13}$$

$$v_{\mathrm{ALD}} = \frac{V_m \left(\mathrm{FBP} - \frac{\mathrm{DHAP}\,\mathrm{GAP}}{K_{\mathrm{eq}}}\right)}{K_{m,\mathrm{FBP}} \left(\frac{\mathrm{FBP}}{K_{m,\mathrm{FBP}}} + \left(\frac{\mathrm{DHAP}}{K_{m,\mathrm{dhap}}} + 1\right) \left(\frac{\mathrm{GAP}}{K_{m,\mathrm{gap}}} + 1\right)\right)} \tag{14}$$

$$v_{\mathrm{TPI}} = \frac{V_m \left(\mathrm{DHAP} - \frac{\mathrm{GAP}}{K_{\mathrm{eq}}}\right)}{K_{m,\mathrm{dhap}} \left(\frac{\mathrm{DHAP}}{K_{m,\mathrm{dhap}}} + \frac{\mathrm{GAP}}{K_{m,\mathrm{gap}}} + 1\right)} \tag{15}$$

$$v_{\mathrm{GPD}} = \frac{V_m \left(\mathrm{DHAP}\,\mathrm{NADH} - \frac{\mathrm{G3P}\,\mathrm{NAD}}{K_{\mathrm{eq}}}\right)}{K_{m,\mathrm{NADH}}\,K_{m,\mathrm{DHAP}} \left(\frac{\mathrm{NAD}}{K_{m,\mathrm{NAD}}} + \frac{\mathrm{NADH}}{K_{m,\mathrm{NADH}}} + 1\right) \left(\frac{\mathrm{DHAP}}{K_{m,\mathrm{DHAP}}} + \frac{\mathrm{G3P}}{K_{m,\mathrm{G3P}}} + 1\right) \left(\frac{\mathrm{ADP}}{K_{i,\mathrm{ADP}}} + \frac{\mathrm{ATP}}{K_{i,\mathrm{ATP}}} + \frac{\mathrm{FBP}}{K_{i,\mathrm{FBP}}} + 1\right)} \tag{16}$$

$$v_{\mathrm{HOR2}} = \frac{V_m\,\mathrm{G3P}}{K_{m,\mathrm{G3P}}\,\left(\frac{\mathrm{G3P}}{K_{m,\mathrm{G3P}}}+1\right)\,\left(\frac{\mathrm{PI}}{K_{i,\mathrm{PI}}}+1\right)} \tag{17}$$

$$V_{\mathrm{GLYCt}} = K_{\mathrm{GLYCt}}\,(\mathrm{GLYC}-\mathrm{GLYCe}) \tag{18}$$

$$v_{\mathrm{GAPDH}} = \frac{V_m\,\left(\mathrm{GAP\,NAD\,PI}-\frac{\mathrm{BPG\,NADH}}{K_{\mathrm{eq}}}\right)}{K_{m,\pi}\,K_{m,\mathrm{nad}}\,K_{m,\mathrm{gap}}\,\left(\left(\frac{\mathrm{BPG}}{K_{m,\mathrm{bpg}}}+1\right)\left(\frac{\mathrm{NADH}}{K_{m,\mathrm{nadh}}}+1\right)+\left(\frac{\mathrm{GAP}}{K_{m,\mathrm{gap}}}+1\right)\left(\frac{\mathrm{NAD}}{K_{m,\mathrm{nad}}}+1\right)\left(\frac{\mathrm{PI}}{K_{m,\pi}}+1\right)-1\right)} \tag{19}$$

$$v_{\mathrm{PGK}} = \frac{V_m\,(\mathrm{ADP\,BPG}\,K_{\mathrm{eq}}-\mathrm{ATP\,P3G})}{K_{m,\mathrm{P3G}}\,K_{m,\mathrm{ATP}}\,\left(\frac{\mathrm{BPG}}{K_{m,\mathrm{BPG}}}+\frac{\mathrm{P3G}}{K_{m,\mathrm{P3G}}}+1\right)\,\left(\frac{\mathrm{ADP}}{K_{m,\mathrm{ADP}}}+\frac{\mathrm{ATP}}{K_{m,\mathrm{ATP}}}+1\right)} \tag{20}$$

$$v_{\mathrm{PGM}} = \frac{V_m\,\left(\mathrm{P3G}-\frac{\mathrm{P2G}}{K_{\mathrm{eq}}}\right)}{K_{m,\mathrm{P3G}}\,\left(\frac{\mathrm{P2G}}{K_{m,\mathrm{P2G}}}+\frac{\mathrm{P3G}}{K_{m,\mathrm{P3G}}}+1\right)} \tag{21}$$

$$v_{\mathrm{ENO}} = \frac{V_m\,\left(\mathrm{P2G}-\frac{\mathrm{PEP}}{K_{\mathrm{eq}}}\right)}{K_{m,\mathrm{P2G}}\,\left(\frac{\mathrm{P2G}}{K_{m,\mathrm{P2G}}}+\frac{\mathrm{PEP}}{K_{m,\mathrm{PEP}}}+1\right)} \tag{22}$$

$$v_{\mathrm{PYK}} = \frac{V_m\,\mathrm{ADP\,PEP}\,\left(\frac{\mathrm{PEP}}{K_{m,\mathrm{pep}}}+1\right)^{\mathrm{hill}}}{K_{m,\mathrm{adp}}\,K_{m,\mathrm{pep}}\,\left(\left(\frac{\mathrm{PEP}}{K_{m,\mathrm{pep}}}+1\right)^{\mathrm{hill}}+L\left(\frac{\frac{\mathrm{ATP}}{K_{i,\mathrm{ATP}}}+1}{\frac{\mathrm{FBP}}{K_{a,\mathrm{FBP}}}+1}\right)^{\mathrm{hill}}\right)\left(\frac{\mathrm{ADP}}{K_{m,\mathrm{adp}}}+1\right)\left(\frac{\mathrm{PEP}}{K_{m,\mathrm{pep}}}+1\right)} \tag{23}$$

$$v_{\mathrm{PDC}} = \frac{V_m\,\left(\frac{\mathrm{PYR}}{K_{m,\mathrm{PYR}}}\right)^{\mathrm{hill}}}{\left(\frac{\mathrm{PYR}}{K_{m,\mathrm{PYR}}}\right)^{\mathrm{hill}}+1} \tag{24}$$

$$v_{\mathrm{ADH}} = -\frac{V_m\,\mathrm{ADH}_{\mathrm{cor}}\left(\mathrm{ETOH}\,\mathrm{NAD} - \frac{\mathrm{ACE}\,\mathrm{NADH}}{K_{\mathrm{eq}}}\right)}{K_{i,\mathrm{NAD}}\,K_{m,\mathrm{ETOH}}\left(\frac{\mathrm{NAD}}{K_{i,\mathrm{NAD}}} + \frac{\mathrm{NADH}}{K_{i,\mathrm{NADH}}} + \frac{\mathrm{ACE}\,K_{m,\mathrm{NADH}}}{K_{i,\mathrm{NADH}}\,K_{m,\mathrm{ACE}}} + \frac{\mathrm{ACE}\,\mathrm{NADH}}{K_{i,\mathrm{NADH}}\,K_{m,\mathrm{ACE}}} + \frac{\mathrm{ETOH}\,K_{m,\mathrm{NAD}}}{K_{i,\mathrm{NAD}}\,K_{m,\mathrm{ETOH}}} + \frac{\mathrm{ETOH}\,\mathrm{NAD}}{K_{i,\mathrm{NAD}}\,K_{m,\mathrm{ETOH}}} + \frac{\mathrm{ACE}\,\mathrm{ETOH}\,\mathrm{NAD}}{K_{i,\mathrm{ACE}}\,K_{i,\mathrm{NAD}}\,K_{m,\mathrm{ETOH}}} + \frac{\mathrm{ACE}\,\mathrm{ETOH}\,\mathrm{NADH}}{K_{i,\mathrm{NADH}}\,K_{i,\mathrm{ETOH}}\,K_{m,\mathrm{ACE}}} + \frac{\mathrm{ACE}\,K_{m,\mathrm{NADH}}\,\mathrm{NAD}}{K_{i,\mathrm{NAD}}\,K_{i,\mathrm{NADH}}\,K_{m,\mathrm{ACE}}} + \frac{\mathrm{ETOH}\,K_{m,\mathrm{NAD}}\,\mathrm{NADH}}{K_{i,\mathrm{NAD}}\,K_{i,\mathrm{NADH}}\,K_{m,\mathrm{ETOH}}} + 1\right)} \tag{25}$$

$$V_{\mathrm{ETOHt}} = K_{\mathrm{ETOHt}}\,(\mathrm{ETOH} - \mathrm{ETOHe}) \tag{26}$$

$$V_{\mathrm{mito}} = \frac{V_m\,\mathrm{ADP}\,\mathrm{PI}}{(\mathrm{ADP} + K_{m,\mathrm{ADP}})\,(K_{m,\mathrm{PI}} + \mathrm{PI})} \tag{27}$$

$$V_{\mathrm{ATPase}} = \frac{\mathrm{ATP}\,K}{\mathrm{ADP}} \tag{28}$$

$$V_{\mathrm{ADK}} = K\left(\mathrm{ADP}^2 - \frac{\mathrm{AMP}\,\mathrm{ATP}}{K_{\mathrm{eq}}}\right) \tag{29}$$

$$V_{\mathrm{vacPi}} = -K\,(\mathrm{PI} - \mathrm{PIvac}) \tag{30}$$

$$V_{\mathrm{mitoNADH}} = \frac{V_m\,\mathrm{NADH}}{K_m + \mathrm{NADH}} \tag{31}$$

$$V_{\mathrm{sinkG6P}} = \frac{V_m\,\mathrm{G6P}}{\mathrm{G6P} + K_m} \tag{32}$$

$$V_{\mathrm{sinkF6P}} = \frac{V_m\,\mathrm{F6P}}{\mathrm{F6P} + K_m} \tag{33}$$

$$V_{\mathrm{sinkGAP}} = \frac{V_m\,\mathrm{GAP}}{\mathrm{GAP} + K_m} \tag{34}$$

$$V_{\mathrm{sinkP3G}} = \frac{V_m\,\mathrm{P3G}}{K_m + \mathrm{P3G}} \tag{35}$$

$$V_{\mathrm{sinkPEP}} = \frac{V_m\,\mathrm{PEP}}{K_m + \mathrm{PEP}} \tag{36}$$

$$V_{\mathrm{sinkPYR}} = \frac{V_m\,\mathrm{PYR}}{K_m + \mathrm{PYR}} \tag{37}$$

$$V_{\mathrm{sinkACE}} = \frac{V_m\,\mathrm{ACE}}{\mathrm{ACE} + K_m} \tag{38}$$

Reactions added in this model:

$$v_{\mathrm{ATH}} = \frac{\mathrm{ATH}\,K_{\mathrm{cat}}\,\mathrm{TRE}}{K_{M,\mathrm{TRE}}\left(\frac{\mathrm{TRE}}{K_{M,\mathrm{TRE}}} + 1\right)} \tag{39}$$

$$v_{\mathrm{AGT1}} = \frac{\mathrm{AGT}_1\,K_{\mathrm{cat}}\left(\mathrm{TRE}_{\mathrm{cyt}} - \frac{\mathrm{TRE}_{\mathrm{ec}}}{K_{\mathrm{eq}}}\right)}{K_{M,\mathrm{TRE}}\left(\frac{\mathrm{TRE}_{\mathrm{ec}}}{K_{M,\mathrm{TRE}}} + \frac{\mathrm{TRE}_{\mathrm{cyt}}}{K_{M,\mathrm{TRE}}} + \frac{\mathrm{UDP}_{\mathrm{GLC}}}{K_{i,\mathrm{UDPglc}}} + 1\right)} \tag{40}$$

$$v_{\mathrm{vacuoleT}} = \frac{V_{\mathrm{max}}\left(\mathrm{TRE}_{\mathrm{cyt}} - \frac{\mathrm{TRE}_{\mathrm{vac}}}{K_{\mathrm{eq}}}\right)}{K_{M,\mathrm{TRE}}\left(\frac{\mathrm{TRE}_{\mathrm{vac}}}{K_{M,\mathrm{TRE}}} + \frac{\mathrm{TRE}_{\mathrm{cyt}}}{K_{M,\mathrm{TRE}}} + 1\right)} \tag{41}$$

Glycogen synthesis and degradation were directly interpolated from the data, with the addition of a saturation component, to avoid that it become a constraint in the system, especially when fluxes were overall small.

$$v_{\mathrm{glyc,synthesis}} = \frac{v_{\mathrm{glyc,synthesis,interpolated}}\,\mathrm{UDP}_{\mathrm{GLC}}}{\mathrm{UDP}_{\mathrm{GLC}} + 1\mathrm{E}^{-4}} \tag{42}$$

$$v_{\mathrm{glyc,degradation}} = \frac{v_{\mathrm{glyc,degradation,interpolated}}\,\mathrm{Glycogen}}{\mathrm{Glycogen} + 1\mathrm{E}^{-4}} \tag{43}$$
