# Supplementary material for: Using Kinetic Modelling to Infer Adaptations in Saccharomyces cerevisiae Carbohydrate Storage Metabolism to Dynamic Substrate Conditions
Source: Metabolites. 2023 Jan 5;13(1):88. doi: 10.3390/metabo13010088 (PMC9862193; doi:10.3390/metabo13010088)

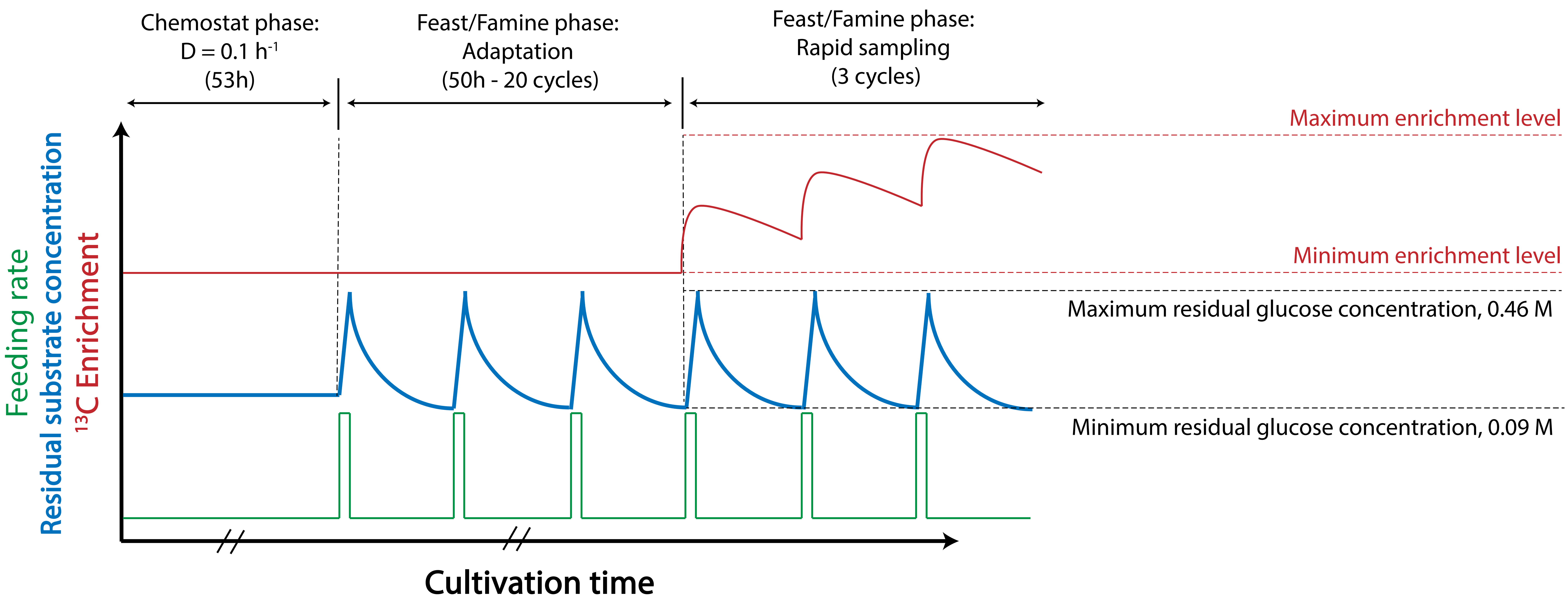

<u>Chemostat phase</u>

$Fin = 1.0817 \cdot 10^{-4}$ $(L\ s^{-1})$

$Fin = Fout$

$Cx = 3.639$ $(g_{DW}\ L^{-1})$

<u>Feeding phase (0-20s)</u>

$Fin = 2.2 \cdot 10^{-3}$, $Fout = 0$ $(L\ s^{-1})$

<u>No feeding phase (20-280s)</u>

$Fin = 0$, $Fout = 1.7 \cdot 10^{-4}$ $(L\ s^{-1})$

<u>No feeding phase (280-400s)</u>

$Fin = 0$, $Fout = 0$ $(L\ s^{-1})$

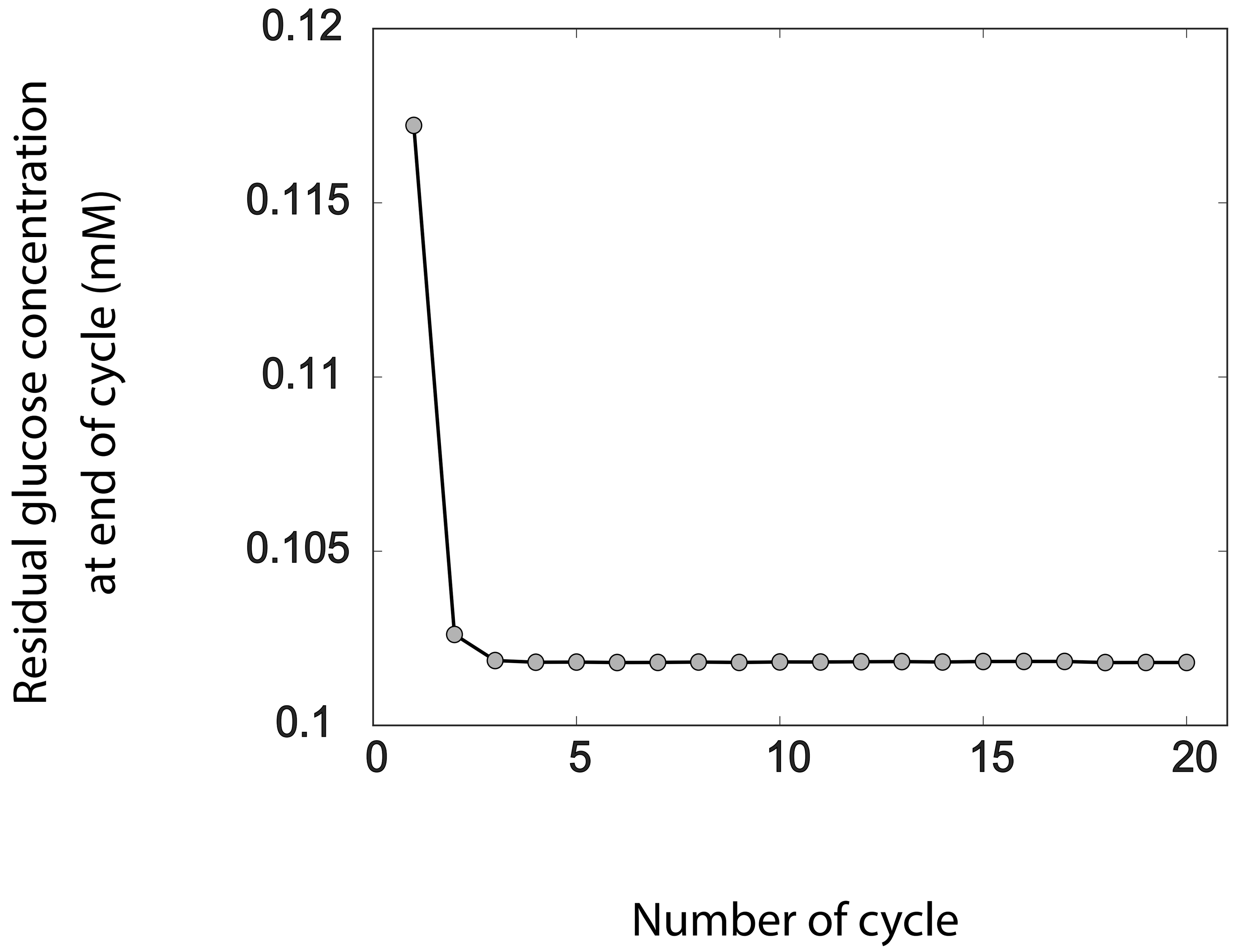

Supplement: Supplementary file 1 [file metabolites-13-00088-s001.zip › metabolites-1971060-supplementary/supplementary_materials/File S1/Simulation setup.pdf]
