# Supplementary material for: Using Kinetic Modelling to Infer Adaptations in Saccharomyces cerevisiae Carbohydrate Storage Metabolism to Dynamic Substrate Conditions
Source: Metabolites. 2023 Jan 5;13(1):88. doi: 10.3390/metabo13010088 (PMC9862193; doi:10.3390/metabo13010088)
Supplement: Supplementary file 1 [file metabolites-13-00088-s001.zip › metabolites-1971060-supplementary/supplementary_materials/S2_Fig.pdf]

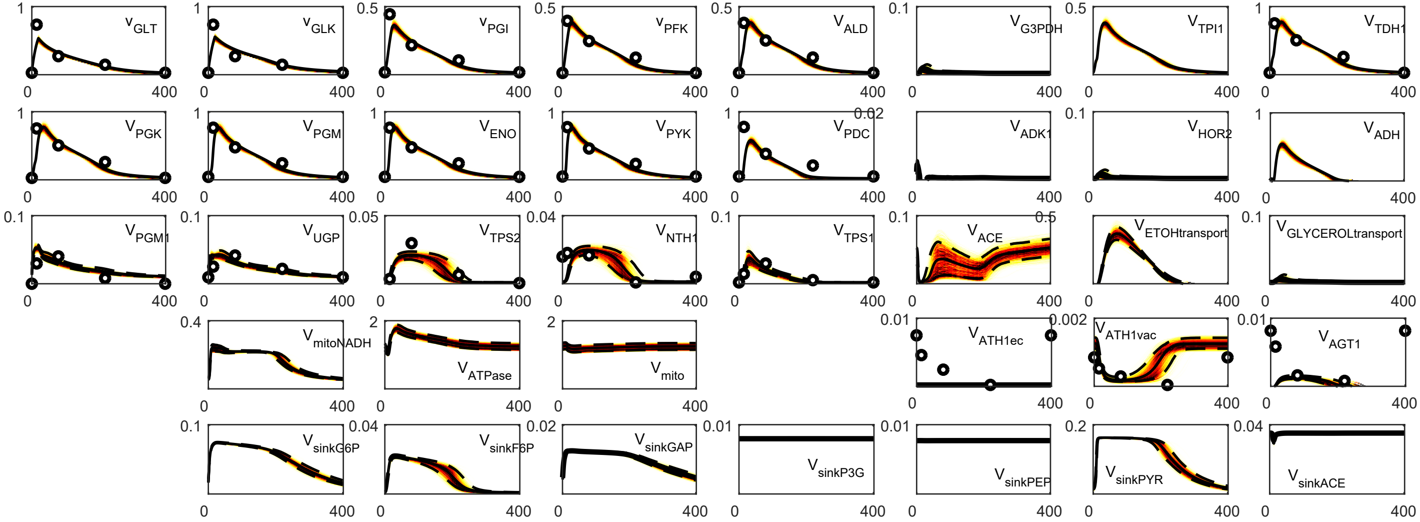

Figure S2. Simulation of reaction rates is robust to parameter changes within 10% of their estimated value.
2D histogram plot of 10.000 model samples. Darker areas point at more dense regions. Models were sampled by adding random noise to all the parameters in the network, within a range of ±10% of the original parameter value.
