# Supplementary material for: Using Kinetic Modelling to Infer Adaptations in Saccharomyces cerevisiae Carbohydrate Storage Metabolism to Dynamic Substrate Conditions
Source: Metabolites. 2023 Jan 5;13(1):88. doi: 10.3390/metabo13010088 (PMC9862193; doi:10.3390/metabo13010088)
Supplement: Supplementary file 1 [file metabolites-13-00088-s001.zip › metabolites-1971060-supplementary/supplementary_materials/S4_Fig.pdf]

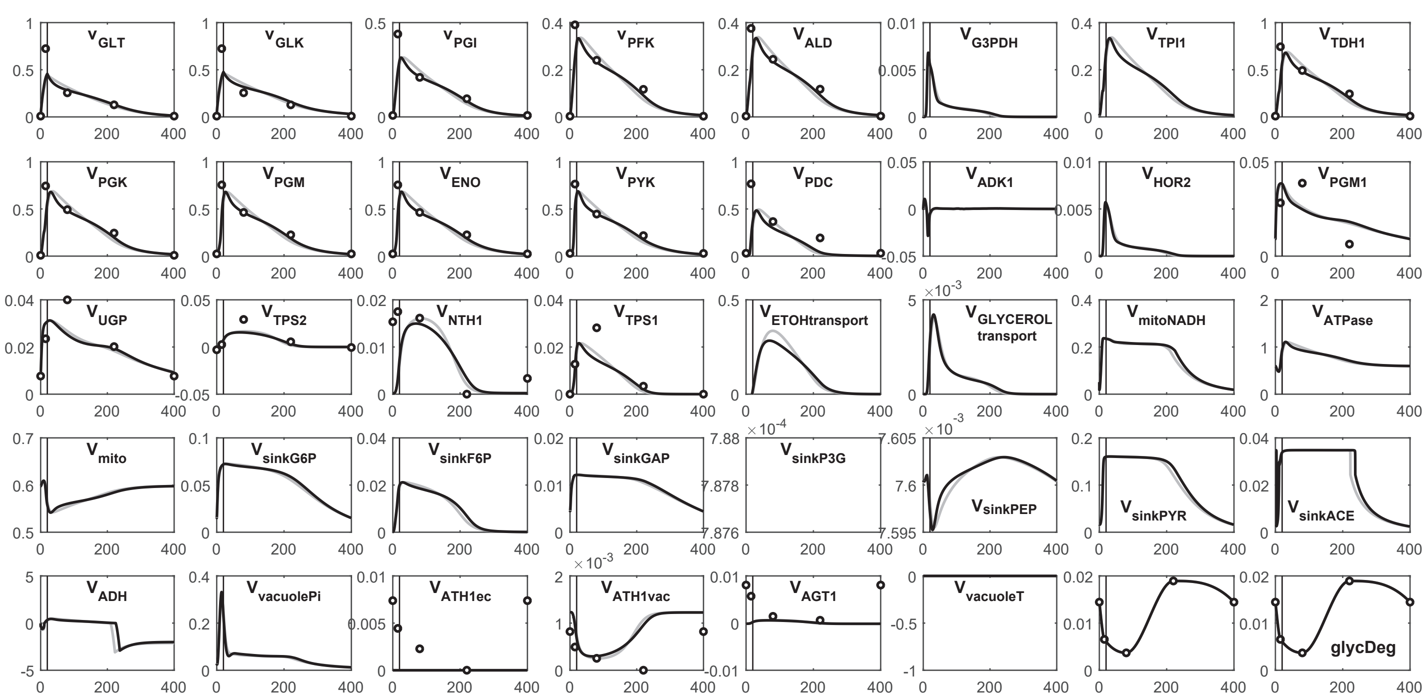

Figure S4. Reaction rates (mM s^{-1}) over time (s). Model fit.
(Gray) Non-regularized, (black) regularized and (black dots) experimental data.
